# Racial and Ethnic Differences in Parental Attitudes and Concerns About School Reopening During the COVID-19 Pandemic — United States, July 2020

**DOI:** 10.15585/mmwr.mm6949a2

**Published:** 2020-12-11

**Authors:** Leah K. Gilbert, Tara W. Strine, Leigh E. Szucs, Tamara N. Crawford, Sharyn E. Parks, Danielle T. Barradas, Rashid Njai, Jean Y. Ko

**Affiliations:** 1 CDC COVID-19 Response Team.

In light of the disproportionate risk of hospitalization and death attributable to coronavirus disease 2019 (COVID-19) among racial and ethnic minority groups, parental attitudes and concerns regarding school reopening were assessed by race and ethnicity using data from three online CARAVAN omnibus surveys conducted during July 8–12, 2020, by ENGINE Insights.* Survey participants included 858 parents who had children and adolescents in kindergarten through grade 12 (school-aged children) living in their household. Overall, 56.5% of parents strongly or somewhat agreed that school should reopen this fall, with some differences by race/ethnicity: compared with 62.3% of non-Hispanic White (White) parents, 46.0% of non-Hispanic Black or African American (Black) parents (p = 0.007) and 50.2% of Hispanic parents (p = 0.014) agreed that school should reopen this fall. Fewer White parents (62.5%) than Hispanic (79.5%, p = 0.026) and non-Hispanic parents of other racial/ethnic groups (66.9%, p = 0.041) were supportive of a mask mandate for students and staff members. Understanding parental attitudes and concerns is critical to informing communication and messaging around COVID-19 mitigation. Families’ concerns also highlight the need for flexible education plans and equitable resource provision so that youth education is not compromised.

Sustained physical proximity and high contact between children and adolescents attending school might increase risk for infection and community and intrahousehold spread of COVID-19, which is associated with worse outcomes for racial and ethnic minority groups (*1*–*3*). Compared with White persons, non-Hispanic American Indian or Alaska Native, Black, non-Hispanic Asian (Asian), and Hispanic persons experience higher COVID-19 incidence, related hospitalizations, and death.^†^ As an important component of community infrastructure, in addition to education, schools provide critical services that help mitigate health disparities, including school meal programs and social, physical, behavioral, and mental health services. COVID-19–related school closures disrupt the delivery of critical services to school-aged children and families and might exacerbate the inequalities faced by racial and ethnic minority families (*4*–*6*). To inform communication and behavior change strategies aimed at COVID-19 mitigation in school settings and to help school districts respond to families’ needs, parental attitudes and concerns about school reopening during the COVID-19 pandemic were assessed.

Data from three online CARAVAN omnibus national surveys conducted among U.S. adults aged ≥18 years during July 8–12 by ENGINE Insights were analyzed. Each survey included approximately 1,000 adults. Quota sampling was conducted by ENGINE Insights to select respondents, and statistical weighting was used during analysis to match the 2019 edition of the Current Population Survey proportions, so the sample represented the U.S. population by sex, age, region, race/ethnicity, and education.^§^ Participants came from the Lucid Marketplace (https://luc.id/quality/) and could opt-in to supplier panels. Incentives were typically offered as points, which could be redeemed for gift cards or prizes. Respondents were eligible if they had not participated in the previous 20 survey administration waves. Respondents were informed that their answers were being used for market research and that they could refuse to answer any question. Data quality filters in the survey prevent multiple responses from the same person or household and improve response completeness. The survey was only administered in English. This activity was reviewed by CDC and was conducted consistent with applicable federal law and CDC policy.^¶^

Among 3,010 respondents, 858 (29%) parents with school-aged children living in the household were included in the analysis. All 858 parents responded to the questions analyzed in this report. Parents were asked about their attitudes and concerns regarding school reopening. Weighted response percentages, p-values, and 95% confidence intervals were calculated, overall and by race/ethnicity. Unadjusted weighted logistic regression was used to test for differences in responses between racial/ethnic groups; differences were considered statistically significant if p-values were ≤0.05. SAS (version 9.4; SAS Institute) was used for all analyses.

Among 858 parent respondents with school-aged children living in the household, 51.1% were women; 55.6% were White, 13.2% were Black, 24.4% were Hispanic, and 6.7% were non-Hispanic, other race (including American Indian or Alaska Native [1%], Asian [4.0%], multiracial [0.9%], and other [0.9%]) (Table 1). Approximately one half of respondents (50.2%) had children in kindergarten through grade 4; 46.1% had children in grades 5–8, and 35.6% had children in grades 9–12. Overall, 248 (29%) respondents selected more than one category for children’s school grade; however, some respondents might have had more than one child in the same grade range. Thus, the total number of respondents with more than one child is not known. In terms of education, 38.0% of parent respondents held less than a high school education, 20.4% had some college or technical school education, and 41.6% held a bachelor’s degree or higher. When looking at household region, 41.1% of parent respondents lived in the South, 23.6% in the West, 19.9% in the Midwest, and 15.4% in the Northeast.

**TABLE 1:** Characteristics of respondents with school-aged children (kindergarten [K]–grade 12) — ENGINE Insights, United States, 2020

Characteristic	Unweighted no. (weighted %)
**Total**	**858 (100)**
**Parent’s race/ethnicity**	
White, non-Hispanic	571 (55.6)
Black, non-Hispanic	88 (13.2)
Hispanic, non-Hispanic	140 (24.4)
Other, non-Hispanic *	59 (6.7)
**Parent’s sex**	
Female	377 (51.1)
**Child's grade level^†^**	
K–4	428 (50.2)
5–8	412 (46.1)
9–12	295 (35.6)
**Household region** ^§^	
Northeast	153 (15.4)
Midwest	160 (19.9)
South	346 (41.1)
West	199 (23.6)
**Education**	
Less than high school	225 (38.0)
Some college or technical school	161 (20.4)
Bachelor’s degree or higher	472 (41.6)

* Other, non-Hispanic includes participants who identified as American Indians and Alaska Natives, Asians, multiracial persons, and other.

^†^ These totals sum to >100% because some parents had more than one school-aged child living in the household.

^§^ *Northeast*: Connecticut, Maine, Massachusetts, New Hampshire, New Jersey, New York, Pennsylvania, Rhode Island, Vermont; *Midwest*: Illinois, Indiana, Iowa, Kansas, Michigan, Minnesota, Missouri, Nebraska, North Dakota, Ohio, South Dakota, Wisconsin; *South*: Alabama, Arkansas, Delaware, District of Columbia, Florida, Georgia, Kentucky, Louisiana, Maryland, Mississippi, North Carolina, Oklahoma, South Carolina, Tennessee, Texas, Virginia, West Virginia; *West*: Alaska, Arizona, California, Colorado, Hawaii, Idaho, Montana, Nevada, New Mexico, Oregon, Utah, Washington, Wyoming.

Compared with White parents, 62.3% of whom strongly or somewhat agreed that schools should reopen in-person for all students in the fall, a smaller percentage of Black (46.0%, p = 0.007) and Hispanic parents (50.2%, p = 0.014) agreed (Table 2). When asked about schooling preferences until a COVID-19 vaccine is available, 82.4% of Hispanic parents strongly or somewhat agreed that they would prefer to homeschool their children until a vaccine is available, compared with 69.8% of White parents (p = 0.006) and 64.7% of parents of other racial/ethnic groups (p = 0.012). Whereas two thirds (67.6%) of White parents agreed that the overall experience of being in school is more important for students, despite ongoing COVID-19 concerns, significantly fewer Hispanic parents (53.9%, p = 0.005) and parents of other racial/ethnic groups (53.4%, p = 0.044) felt this way. Reported concern about students complying with mitigation was significantly higher among parents of other racial/ethnic groups (96.9%) compared with White parents (85.2%, p = 0.025) and Hispanic parents (80.6%, p = 0.009). Similarly, a higher percentage of Black parents (91.9%) were concerned about mitigation compliance than were Hispanic parents (80.6%, p = 0.019).

**TABLE 2:** Parental attitudes and concerns about risks and benefits of school reopening during the COVID-19 pandemic and impacts of COVID–19 on student academic and health-related outcomes, by race/ethnicity — ENGINE Insights, United States, 2020

Question and response	Racial/Ethnic group, % (95% CI)				
	Overall*	White, non-Hispanic*	Black, non-Hispanic*	Hispanic or Latino*	Other,^†^ non-Hispanic*
**How much do you agree or disagree with the following?**					
**Schools should reopen for all students in the fall**					
Strongly/Somewhat agree	56.5 (52.8–60.3)	62.3 (57.7–66.9)	46.0 (35.0–57.0)^§^	50.2 (41.5–58.8)^§^	52.6 (39.1–66.1)
**I would rather homeschool my child until a COVID-19 vaccine is available**					
Strongly/Somewhat agree	73.3 (70.0–76.6)	69.8 (65.5–74.2)	75.6 (66.2–85.0)	82.4 (75.8–88.9)^§^	64.7 (51.6–77.8)^¶^
**The overall experience of being in school is more important for students, despite ongoing COVID-19 concerns around the country**					
Strongly/Somewhat agree	61.8 (58.1–65.5)	67.6 (63.2–72.0)	56.5 (45.4–67.7)	53.9 (45.3–62.5)^§^	53.4 (39.9–67.0)^§^
**Even if measures are put in place, I am concerned about students following through and fully complying with social distancing and mask wearing mandates**					
Strongly/Somewhat agree	85.7 (83.1–88.4)	85.2 (81.9–88.6)	91.9 (86.6–97.2)^¶^	80.6 (73.6–87.5)	96.9 (92.5–100.0)^§,¶^
**Thinking about this upcoming school year, how concerned are you about the following?**					
**The quality of your children’s education being negatively impacted by the COVID–19 pandemic**					
Very/Somewhat concerned	89.4 (87.1–91.8)	90.9 (88.2–93.7)	89.3 (82.9–95.8)	84.9 (78.8–91.0)	93.4 (87.6–99.2)
**School reopening safely in the fall**					
Very/Somewhat concerned	87.8 (85.4–90.3)	86.0 (82.7–89.3)	93.5 (88.6–98.3)^§^	86.0 (80.1–91.8)	98.8 (96.3–100.0)^§,¶^
**The potential disruption to your daily routines if virtual (at–home) learning becomes necessary**					
Very/Somewhat concerned	77.4 (74.1–80.6)	78.6 (74.5–82.5)	78.8 (70.0–87.6)	71.8 (63.9–79.6)	84.7 (75.2–94.2)
**Your child contracting COVID–19 as a result of attending school**					
Very/Somewhat concerned	86.3 (83.7–88.9)	84.1 (80.6–87.6)	92.6 (87.4–97.8)^§^	85.5 (79.4–91.6)	95.6 (90.6–100.0)^§,¶^
**Your child bringing COVID–19 home as a result of attending school**					
Very/Somewhat concerned	86.3 (83.8–88.9)	84.5 (81.0–87.9)	92.7 (87.3–98.2)^§^	86.2 (80.4–92.0)	89.4 (80.9–98.0)

**Abbreviations:** CI = confidence interval; COVID-19 = coronavirus disease 2019.

* Weighted.

^†^ Other, non-Hispanic includes participants who identified as American Indian/Alaska Native, Asian, multiracial, and other.

^§^ p≤0.05 compared with White, non-Hispanic.

^¶^ p≤0.05 compared with Hispanic.

The majority of parents (89.4% overall) were concerned about the quality of their children’s education being negatively affected by the COVID-19 pandemic, with no statistically significant differences between racial and ethnic groups. Parents of other racial/ethnic groups were more likely to be very or somewhat concerned about schools opening safely in the fall (98.8%) than were White (86.0%, p = 0.012) and Hispanic (86.0%, p = 0.014) parents. Black parents were also more likely to be very or somewhat concerned about schools reopening safely in the fall (93.5%) compared with White parents (86.0%, p = 0.049). No statistically significant racial or ethnic differences were observed in feeling concerned about disruption to daily routines if virtual learning were to become necessary (77.4% overall). Parents of other racial/ethnic groups were more likely to be very or somewhat concerned about their child contracting COVID-19 as a result of attending school (95.6%) than were White parents (84.1%, p = 0.023) and Hispanic parents (85.5%, p = 0.047); Black parents were significantly more likely to be concerned about this (92.6%) than were White parents (84.1%, p = 0.036). More Black parents were very or somewhat concerned about their child bringing home COVID-19 from school (92.7%) than were White parents (84.5%, p = 0.050).

Overall, 52.7% of parents were very or somewhat comfortable with their children’s schools opening at full capacity in the fall (Table 3). Parents of other racial/ethnic groups were less likely to be comfortable with their children’s schools opening at full capacity (32.5%) than were White parents (57.1%, p = 0.001) and Hispanic parents (53.3%, p = 0.011); Black parents (43.0%) were less likely to be comfortable than were White parents (57.1%, p = 0.022). Finally, although most parents supported mask mandates, fewer White parents were supportive of a mask mandate for students and staff members (62.5%) than were Hispanic parents (79.5%, p = 0.026) and parents of other racial/ethnic groups (66.9%, p = 0.041).

**TABLE 3:** Parental attitudes and concerns about school reopening strategies and mask mandates, by race/ethnicity — ENGINE Insights, United States, 2020

Questions and responses	Racial/Ethnic group, % (95% CI)				
	Overall*	White, non-Hispanic*	Black, non-Hispanic *	Hispanic or Latino*	Other,^†^ non-Hispanic*
**In light of the COVID-19 pandemic, how comfortable would you be with the following:**					
**Your children’s school(s) reopening at full capacity in the fall**					
Very comfortable/Somewhat comfortable	52.7 (48.9–56.4)	57.1 (52.4–61.8)	43.0 (32.0–53.9)^§^	53.3 (44.7–61.9)	32.5 (20.1–44.9) ^§,¶^
**Your children’s school(s) reopening at 50% capacity in the fall, with the other 50% dedicated to virtual learning**					
Very comfortable/Somewhat comfortable	66.2 (62.6–69.8)	67.9 (63.5–72.4)	58.2 (47.1–69.3)	67.1 (59.0–75.2)	64.8 (52.1–77.6)
**Your children’s school(s) reopening in the fall exclusively with virtual learning**					
Very comfortable/Somewhat comfortable	69.7 (66.2–73.2)	69.1 (64.7–73.6)	73.3 (63.7–82.9)	69.8 (61.8–77.9)	66.7 (53.9–79.6)
**When school resumes in the fall, do you believe wearing masks/facial coverings should be mandated for everyone (both students and staff)?**					
Yes, at all times	68.3 (64.8–71.8)	62.5 (57.9–67.1)	73.1 (63.4–82.7)	79.5 (72.7–86.4)^§^	66.9 (54.2–79.5)^§^

**Abbreviations:** CI = confidence interval; COVID-19 = coronavirus disease 2019.

* Weighted.

^†^ Other, non-Hispanic includes participants who identified as American Indian/Alaska Native, Asian, multiracial, and other.

^§^ p≤0.05 compared with White, non-Hispanic.

^¶^ p≤0.05 compared with Hispanic.

## Discussion

Although the majority of parent respondents had concerns about both school reopening for in-person instruction and virtual learning, the perceived risk for SARS-CoV-2 infection and poor health outcomes might account for the differences in parental attitudes and concerns by race and ethnicity (*7*). Compared with White parents, non-White parents were less likely to feel that schools should reopen for all students and were more concerned about adherence to mitigation strategies, schools reopening safely, their child contracting COVID-19, and their child bringing home COVID-19.

Existing structural inequalities place racial and ethnic minority groups at increased risk for poor health outcomes, and social determinants of health, such as discrimination, health care access and utilization, occupation, education, income and wealth gaps, and housing, contribute to the disproportionate rates of COVID-19 incidence morbidity and associated hospitalization and mortality rates.** Further, socioeconomically disadvantaged families, including those in racial and ethnic minority populations, and those residing in rural areas, might have fewer resources available to support remote learning, including high-speed Internet access, computers, and job flexibility (*7*,*8*). In addition, family structure (e.g., number of siblings or other relatives in the household) and the ability to find alternative sources of child care might influence parental attitudes and concerns. However, the fear of poor health outcomes from COVID-19 might outweigh these obstacles as families make choices about in-person or virtual learning.

The current school year is well underway; however, these findings remain relevant as the pandemic evolves and families and school districts continue to weigh the risks and benefits of in-person versus virtual instruction. School districts should be cognizant of medical risks for severe COVID-19 and resource limitations among families while also considering their own resources to successfully implement mitigation strategies and provide flexibility in their approach to schooling.

Community mitigation efforts can only succeed if they are supported by the community. A smaller percentage of White parents were supportive of mask mandates than were Hispanic parents and parents of other racial/ethnic groups; these findings are consistent with earlier studies, which have found lower adherence to mask-wearing recommendations and mandates among White adults (*9*). Messages about the importance of wearing masks in public spaces or reassurance that following mitigation measures appropriately can prevent COVID-19 might need to be tailored to different community groups (*10*).

The findings in this report are subject to at least six limitations. First, data were self-reported; therefore, responses might be subject to social desirability bias. Second, although survey responses were weighted to be nationally representative of U.S. demographics, whether responses among this incentivized, opt-in panel sample are truly representative of attitudes and concerns shared by the broader U.S. population or what biases might have occurred is not known. Third, responses were recorded at a single point in time and might not reflect shifts in parental attitudes and concerns about school opening in light of varying community transmission rates and learning options. Fourth, because some families might have more than one child, these questions might have been difficult to answer if concern varied by the child’s age and school environment. Fifth, the sample could be biased because the survey was only administered in English. Finally, because of sample size, this study did not adjust for other factors, such as socioeconomic status, urbanicity, or geographic region, which might also affect parental attitudes and concerns.

As U.S. schools make decisions about in-person or virtual learning, ongoing monitoring of parental concerns about school reopening and virtual learning is critical to ensure that families are getting the support they need. Community mitigation implementation and compliance in the school setting should be maximized to reduce COVID-19 transmission.

**Summary**

**What is already known about this topic?**

Families and school districts face challenges balancing COVID-19 mitigation and school reopening.

**What is added by this report?**

Among parents of school-aged children who participated in an Internet panel survey, racial and ethnic minority parents were more concerned about some aspects of school reopening, such as compliance with mitigation measures, safety, and their child contracting or bringing home COVID-19, than were non-Hispanic White parents.

**What are the implications for public health practice?**

Understanding racial/ethnic differences in parental attitudes and concerns about school reopening can inform communication and mitigation strategies and highlights the importance of considering risks for severe COVID-19 and family resource needs when developing options for school attendance during the COVID-19 pandemic.

## Footnotes

* https://engine-insights.com/product/caravan/.

† https://www.cdc.gov/coronavirus/2019-ncov/covid-data/investigations-discovery/hospitalization-death-by-race-ethnicity.html.

§
https://www.census.gov/programs-surveys/cps.html .

¶ 45 C.F.R. part 46; 21 C.F.R. part 56; 42 U.S.C. Sect. 241(d), 5 U.S.C. Sect. 552a, 44 U.S.C. Sect. 3501 et seq.

** https://www.healthypeople.gov/2020/topics-objectives/topic/social-determinants-of-health.
